# Headache severity in patients with post COVID-19 condition: a case-control study

**DOI:** 10.1007/s00406-024-01850-8

**Published:** 2024-06-25

**Authors:** Mike Rueb, Michael Ruzicka, Gerardo Jesus Ibarra Fonseca, Elisabeth Valdinoci, Christopher Benesch, Anna Pernpruner, Max von Baum, Jan Remi, Tarek Jebrini, Florian Schöberl, Andreas Straube, Hans Christian Stubbe, Kristina Adorjan

**Affiliations:** 1 Department of Psychiatry and Psychotherapy, LMU University Hospital, LMU Munich, Nußbaumstraße 7, 80336 Munich, Germany; 2 Pettenkofer School of Public Health, Munich, Germany; 3 Institute for Medical Information Processing, Biometry and Epidemiology, LMU University Hospital, LMU Munich, Munich, Germany; 4 Center for International Health (CIH LMU), LMU University Hospital, LMU Munich, Munich, Germany; 5 Institute of Medical Education, LMU University Hospital, LMU Munich, Munich, Germany; 6 Department of Medicine III, LMU University Hospital, LMU Munich, Munich, Germany; 7 Department of Medicine IV, LMU University Hospital, LMU Munich, Munich, Germany; 8 Department of Neurology, LMU University Hospital, LMU Munich, Munich, Germany; 9 Department of Medicine II, LMU University Hospital, LMU Munich, Munich, Germany; 10 German Centre for Infection Research (DZIF), Partner Site Munich, Munich, Germany; 11 Institute of Psychiatric Phenomics and Genomics, LMU University Hospital, Munich, Germany; 12 University Hospital of Psychiatry and Psychotherapy, University of Bern, Bern, Switzerland

**Keywords:** Post COVID-19 condition, Post COVID-19 syndrome, Post COVID, Long COVID, Headache, Quality of life

## Abstract

Post COVID-19 conditions (PCC) present with a wide range of symptoms. Headache is one of the most frequently reported neurological symptoms by patients with PCC. We aimed to assess the prevalence of headache in patients with PCC who attended the Post-COVID^LMU^ outpatient department at LMU University Hospital in Munich. We hypothesized that headaches occur more frequently in patients with PCC than in the control group. Patients answered a questionnaire containing sociodemographic characteristics, their current symptoms, and prior psychiatric and somatic diagnoses, the WHO Quality of Life assessment (WHOQOL-BREF), 9-item Patient Health Questionnaire (PHQ-9), and the Fatigue Severity Scale (FSS). 188 PCC patients were included in this study and compared to a control group of patients with a history of COVID-19 or a different infectious disease – but no consecutive post-infectious condition (n_c_=27). 115 (61%) of our PCC patients were female. The median age was 41 years. 60 (32%, *p* = 0.001) had a pre-existing psychiatric diagnosis. PCC was associated with worse outcomes in all four domains of the WHOQOL-BREF (*p* < 0.001), high levels of fatigue (FSS; *p* < 0.001), and a higher likeliness for symptoms of depression (PHQ-9; *p* < 0.001). We were able to confirm that psychiatric disorders are more frequently associated with headaches in PCC patients. Headache should be assessed and treated in the context of PCC not only by neurologists but by multi-professional teams and regarding all PCC symptoms.

**Supplementary Information:**

The online version contains supplementary material available at 10.1007/s00406-024-01850-8.

## Introduction

Post COVID-19 conditions (PCC) are a constellation of symptoms that persist beyond the acute phase of COVID-19, often for weeks or months after the initial infection with severe acute respiratory syndrome coronavirus type 2 (SARS-CoV-2) [1–3]. PCC may affect various organ systems and present with a wide range of symptoms (e.g. pulmonary, cardiovascular, immunological, musculoskeletal, psychiatric, and neurological) [3–5]. The condition is associated with a low quality of life [6]. The World Health Organization (WHO) defines PCC as a continuation or development of new symptoms within three months after the initial SARS-CoV-2 infection, with a minimum symptom duration of two months [7]. The exact prevalence and incidence of PCC vary across the published literature. WHO suggests that it affects a substantial proportion of COVID-19 survivors, ranging from 10 to 20% [7]. The United States Centers for Disease Control and Prevention estimated the prevalence of post-COVID-19 conditions between 5 and 30% [8].

Headache is one of the most commonly reported symptoms in patients with PCC [9–11]. Headache has emerged as a common complaint, affecting a significant proportion of patients with PCC [6]. Neurological symptoms are associated with higher severity of PCC [12]. Among PCC patients, headache belongs to the most frequently reported neurological symptoms [9, 13]. Post-viral headache is an acknowledged phenomenon [14, 15]. Large meta-analyses conclude that the prevalence of headaches in the context of PCC ranges from 8 to 44% [6, 16–18]. PCC patients can experience headaches as a disabling condition [19]. The prevalence of headache is associated with more severe courses of COVID-19. Patients hospitalized with COVID-19 were at highest risk of developing PCC associated headache [20, 21].

The manifestation of headache during the acute phase of COVID-19 represents a significant risk factor for persistent headache in the sense of a PCC. Conversely, there appears to be no apparent association between a pre-existing history of primary headache and the occurrence of post-COVID headache [21].

The clinical impact of headaches in PCC patients transcends mere discomfort, manifesting as a substantive detriment to patients’ quality of life and impeding their resumption of routine activities. Additionally, these headaches have been implicated in contributing to mental health disturbances [22]. The diverse spectrum of headache presentations observed within the PCC population underscores the clinically challenging nature of this phenomenon. Patients may exhibit tension-type headaches, migraines, or atypical headache patterns. Noteworthy is the persistence of headaches initiated during the acute COVID-19 phase in some individuals, while others manifest de novo headache [23]. Discerning this variability is imperative for tailoring treatment modalities and optimizing overall patient outcomes [24].

The pathophysiology of headache in PCC is not fully understood, but it is likely to be multifactorial. The specific pathophysiological mechanisms causing PCC symptoms remain elusive and current evidence suggests it’s more indicative of an inflammatory reaction [25, 26]. One hypothesis suggests that elevated glutamate levels and upregulation of N-methyl-D-aspartate (NMDA) receptors might be responsible for symptoms like headaches [27, 28]. PCC headache may be associated with sustained inflammation and hyperimmune activity, as indicated by elevated serum levels of interleukin 6 and 10 (IL-6, IL-10) and inflammasome components. [28, 29] A psychosomatic origin of headaches in the context of PCC also seems plausible [13].

We aimed to assess the prevalence of headache in patients with PCC who attended the Post-COVID^LMU^ outpatient department at the Ludwig Maximilian University (LMU) Hospital [30]. We hypothesized that headaches occur more frequently in PCC than in the control group, and further sought to identify risk factors for developing PCC associated headaches.

## Materials and methods

### Setting

At LMU Hospital in Munich, Germany, the interdisciplinary and cross-sectoral healthcare and research network Post-COVID^LMU^ outpatient department was responsible for diagnosing and providing interdisciplinary treatment for complex and severe post-COVID-19 (i.e. multiple organ systems affected simultaneously, significant deterioration over time, emergence of new symptoms during the course) adult patients [31]. Referrals to the department and appointments were exclusively made through a primary care physician or a specialist. The patient care for this specific group involved close collaboration with numerous specialized departments at LMU, ensuring comprehensive and interdisciplinary involvement. Each patient was evaluated with the involvement of infectious diseases, pulmonology, cardiology, psychiatry and psychosomatics, neurology, physical medicine, and pain therapy departments as required. The treatment services included telemedical consultations and interdisciplinary case conferences, allowing referring physicians to participate actively [30].

Patients who received medical care at our Post-COVID^LMU^ outpatient department were included in the Post-COVID-Care Study (DRKS00030974) upon written informed consent, if they met the criteria for a PCC as defined by the World Health Organization (WHO) [7], and if their initial diagnosis of COVID-19 was confirmed through SARS-CoV-2 PCR testing.

### Measures

For the questionnaire’s items, we used a yes-no option and “unknown” where applicable. Socio-demographic characteristics included sex at birth, age at inclusion, on sick leave, pre-existing psychiatric and somatic diagnoses, and several symptoms (cf. Tables 1 and 2). The patients reported their headache on a four-point Likert scale (no, mild, intermediate, severe headache). In addition, the patients completed three assessments: the 26-item WHO Quality of Life assessment (WHOQOL-BREF) [32, 33], the 9-item Patient Health Questionnaire (PHQ-9) [34, 35], and the Fatigue Severity Scale (FSS) [36, 37]. The WHOQOL-BREF assesses the patients’ quality of life across four domains (physical health, psychological health, social relationships, and environment) using a scale from 0 to 100. Higher scores indicate a better quality of life. To interpret the results the following reference values were used [38]: 73.5 (standard deviation (SD) = 18.1) for physical health, 70.6 (SD = 14.0) for psychological health, 71.5 (SD = 18.2) for social relationships, and 75.1 (SD = 13.0) for environment. The PHQ-9 assesses the severity of depressive disorders on a scale from 0 to 27, allowing screening and grading. The FSS measures fatigue levels on a scale from 1 to 7, with a cut-off of ≥ 5 points indicating a severe fatigue.

### Data analysis

All clinical data were recorded using the Lightweight Clinical Data Acquisition and Management Software for Clinical Research (LCARS-M2, LMU Munich, Germany). The patients and physicians submitted their findings through the LCARS-M2 app, a progressive web app. Each assessment was conducted independently and blinded to the reports from other departments. The relevant clinical data for this study were extracted from the LCARS-M2 database.

Statistical analyses were performed using R Studio version 4.3.0. Numeric variables were presented as median values (Mdn) and the corresponding interquartile ranges (IQR) in brackets. Categorical variables were presented as absolute counts with percentages in brackets. To determine statistical significance between groups, a two-sided Kruskal-Wallis test or Wilcoxon rank sum test was used for numeric variables, while Pearson’s Chi-squared test was employed to assess statistical differences in categorical data. P-values were adjusted for multiple comparisons using the Benjamini-Hochberg procedure where applicable. Statistical significance was considered at values less than 0.05. We visualized the data in balloon plots and a correspondence analysis.

### Ethical approval

Ethical approval for the study was obtained from the Ethics Committee of the Medical Faculty of LMU Munich (21-1165). All potentially eligible participants were informed about the research in oral and written form. Furthermore, participants signed an informed consent form. The Post-COVID-Care study was registered with the *Deutsches Register Klinischer Studien* (DRKS; ‘German Clinical Trials Register’, registration number DRKS00030974).

## Results

### Patient characteristics

In total, 188 patients (n_0_ = 188) with PCC from our Post-COVID^LMU^ outpatient department were included in this study. We compared the PCC patients with a control group (n_c_=27, see Table 1). Patients of the control group were admitted to LMU Hospital due to infectious disease (including a SARS-CoV-2 infection) but did not develop a post-infectious condition. Out of the 188 PCC patients, the majority (*n* = 115, 61%) was female. The median age was 41 (32, 53) years. Ninety-six (54%, *p* < 0.001) of the PCC patients were on sick leave. Sixty (32%, *p* = 0.001) had a psychiatric diagnosis at baseline.

There was a significant reduction in quality of life in PCC compared to the control group in all four domains of the WHOQOL-BREF (*p* < 0.001, Table 1). Regarding the severity of headaches, only the WHOQOL subdomains *physical health* and *environment* showed a significant reduction (*p* < 0.001, Table 2). The Mdn Karnofsky index of 70% (70, 80; *p* < 0.001) indicated an incapacity to engage in work (Table 2).

In the FSS, PCC patients reported an overall score of 6.22 (5.11, 6.67; *p* < 0.001, Table 1), indicating severe fatigue – compared to the control group with a score of 2.35 (1.70, 3.28).

PCC patients scored a median of 10.0 points in the PHQ-9 (7.0, 14.0; *p* < 0.001, Table 1), indicating a moderate depression severity – compared to 1.0 (0.3, 3.0) in the control group.

**Table 1:** Demographic data of the study population

Variable	Control group, *N* = 27^*1*^	Post COVID-19 condition group, *N* = 188^*1*^	*p*-value^2^
**Sex at birth**			> 0.9
Female	17 (63%)	115 (61%)	
Male	10 (37%)	73 (39%)	
**Age at inclusion**	31 (29, 54)	41 (32, 53)	0.2
**Karnowski index**	100 (100, 100)	70 (70, 80)	< 0.001
**On sick leave**			< 0.001
No	19 (83%)	64 (36%)	
Unknown	0 (0%)	19 (11%)	
Yes	4 (17%)	96 (54%)	
**Has psychiatric diagnosis**	0 (0%)	60 (32%)	0.001
**Has somatic diagnosis**	21 (78%)	142 (76%)	> 0.9
**Depressive episode, unspecified (F32.9)**	0 (0%)	33 (18%)	0.037
**Post viral fatigue syndrome [Chronic fatigue syndrome] (G93.3)**	2 (7.4%)	32 (17%)	0.3
**Essential (primary) hypertension (I10)**	3 (11%)	20 (11%)	> 0.9
**Cognitive functional impairment (U51)**	1 (3.7%)	21 (11%)	0.4
**Hyperlipidemia, unspecified (E78.5)**	9 (33%)	11 (5.9%)	< 0.001
**Asthma, unspecified (J45.9)**	3 (11%)	15 (8.0%)	0.9
**Hypothyroidism, unspecified (E03.9)**	1 (3.7%)	17 (9.0%)	0.6
**Autoimmune thyroiditis (E06.3)**	1 (3.7%)	11 (5.9%)	> 0.9
**Anxiety disorder, unspecified (F41.9)**	0 (0%)	12 (6.4%)	0.4
**Type 2 diabetes mellitus (E11)**	2 (7.4%)	7 (3.7%)	0.7
**PRO: WHOQOL-BREF: physical health**	93 (77, 96)	43 (30, 57)	< 0.001
**PRO: WHOQOL-BREF: psychological health**	79 (67, 85)	54 (42, 67)	< 0.001
**PRO: WHOQOL-BREF: social relationship**	83 (75, 83)	67 (50, 75)	< 0.001
**PRO: WHOQOL-BREF: environment**	91 (81, 94)	72 (63, 81)	< 0.001
**PRO: FSS**	2.35 (1.70, 3.28)	6.22 (5.11, 6.67)	< 0.001
**PRO: PHQ-9**	1.0 (0.3, 3.0)	10.0 (7.0, 14.0)	< 0.001

^1^ n (%), Median (IQR); ^*2*^ Pearson’s Chi-squared test, Wilcoxon rank sum test

### Headache severity

Of the 188 patients with PCC, 96 (51%) reported no headache, 27 (14%) a mild headache, 46 (25%) an intermediate headache and 19 (10%) a severe headache (Table 2). 74% of female patients reported intermediate or severe headaches compared to 26% of male patients (*p* = 0.051). Having a pre-existing psychiatric diagnosis was not significantly associated with headache severity (*p* = 0.11). PHQ-9 showed a significant correlation with headache severity (*p* < 0.001).

A significant correlation (*p* < 0.001) was found between the severity of the headache and the following symptoms: dysuria, fever, hematemesis, hypoesthesia, insomnia, joint or muscle pain, taste loss, melena, memory impairment, nausea, night sweats, peripheral edema, red eye, and throat ache (Table 2). Additional data are given in Online Resource 1.

**Table 2:** Symptoms across patients with Post COVID-19 condition with or without headache

Variable	No headache, *N* = 96^*1*^	Mild headache, *N* = 27^*1*^	Intermediate headache, *N* = 46^*1*^	Severe headache, *N* = 19^*1*^	*p*-value^3^	q-value^3^
**Cohort**						
Post COVID-19 condition	96 (100%)	27 (100%)	46 (100%)	19 (100%)		
**Sex at birth**					0.051	0.091
Female	50 (52%)	17 (63%)	34 (74%)	14 (74%)		
Male	46 (48%)	10 (37%)	12 (26%)	5 (26%)		
**Karnofsky index**	70.0 (70.0, 80.0)	70.0 (70.0, 80.0)	70.0 (70.0, 80.0)	70.0 (70.0, 70.0)	0.7	0.7
**Age at inclusion**	42 (32, 53)	37 (30, 48)	41 (32, 52)	41 (33, 54)	0.6	0.7
**Has psychiatric diagnosis**	24 (25%)	8 (30%)	19 (41%)	9 (47%)	0.11	0.2
**Has somatic diagnosis**	74 (77%)	20 (74%)	34 (74%)	14 (74%)	> 0.9	> 0.9
**Type 2 diabetes mellitus (E11)**	3 (3.1%)	1 (3.7%)	3 (6.5%)	0 (0%)	0.6	0.7
**PRO: WHO physical health**	46 (36, 68)	46 (32, 54)	39 (29, 54)	21 (14, 32)	< 0.001	< 0.001
**PRO: WHO psychological health**	63 (45, 71)	54 (46, 67)	52 (45, 63)	46 (33, 54)	0.010	0.026
**PRO: WHO social relationship**	67 (58, 75)	67 (42, 75)	67 (50, 83)	50 (50, 67)	0.12	0.2
**PRO: WHO environment**	75 (67, 84)	69 (59, 81)	72 (66, 78)	56 (50, 63)	< 0.001	0.002
**PRO: FSS**	5.67 (4.67, 6.56)	6.33 (5.44, 6.67)	6.41 (5.44, 7.00)	6.67 (6.44, 6.89)	0.002	0.008
**PRO: PHQ-9**	8.0 (5.0, 13.0)	10.0 (8.0, 13.0)	11.0 (8.0, 15.0)	17.0 (12.8, 19.0)	< 0.001	< 0.001
**Dysuria**	6 (6.3%)	1 (3.7%)	3 (6.5%)	7 (37%)	< 0.001	0.001
**Fever**	3 (3.1%)	2 (7.4%)	1 (2.2%)	5 (26%)	< 0.001	0.003
**Hematemesis**	0 (0%)	0 (0%)	0 (0%)	2 (11%)	< 0.001	0.002
**Hypoesthesia**	7 (7.3%)	4 (15%)	6 (13%)	9 (47%)	< 0.001	< 0.001
**Insomnia**	43 (45%)	19 (70%)	29 (63%)	17 (89%)	< 0.001	0.004
**Joint or muscle pain**	30 (31%)	16 (59%)	23 (50%)	15 (79%)	< 0.001	0.002
**Taste loss/Hypogeusia**	18 (19%)	8 (30%)	15 (33%)	13 (68%)	< 0.001	0.001
**Melena**	0 (0%)	0 (0%)	0 (0%)	3 (16%)	< 0.001	< 0.001
**Memory impairment**	51 (53%)	17 (63%)	34 (74%)	19 (100%)	< 0.001	0.003
**Nausea**	8 (8.3%)	11 (41%)	10 (22%)	10 (53%)	< 0.001	< 0.001
**Night sweats**	23 (24%)	10 (37%)	21 (46%)	14 (74%)	< 0.001	0.001
**Peripheral edema**	4 (4.2%)	1 (3.7%)	5 (11%)	6 (32%)	< 0.001	0.004
**Red eye**	5 (5.2%)	4 (15%)	5 (11%)	8 (42%)	< 0.001	< 0.001
**Throat ache**	5 (5.2%)	6 (22%)	11 (24%)	11 (58%)	< 0.001	< 0.001

^1^ n (%), Median (IQR); ^2^ Pearson’s Chi-squared test, Kruskal-Wallis rank sum test; ^3^ Benjamini & Hochberg correction for multiple testing

**Fig. 1:**
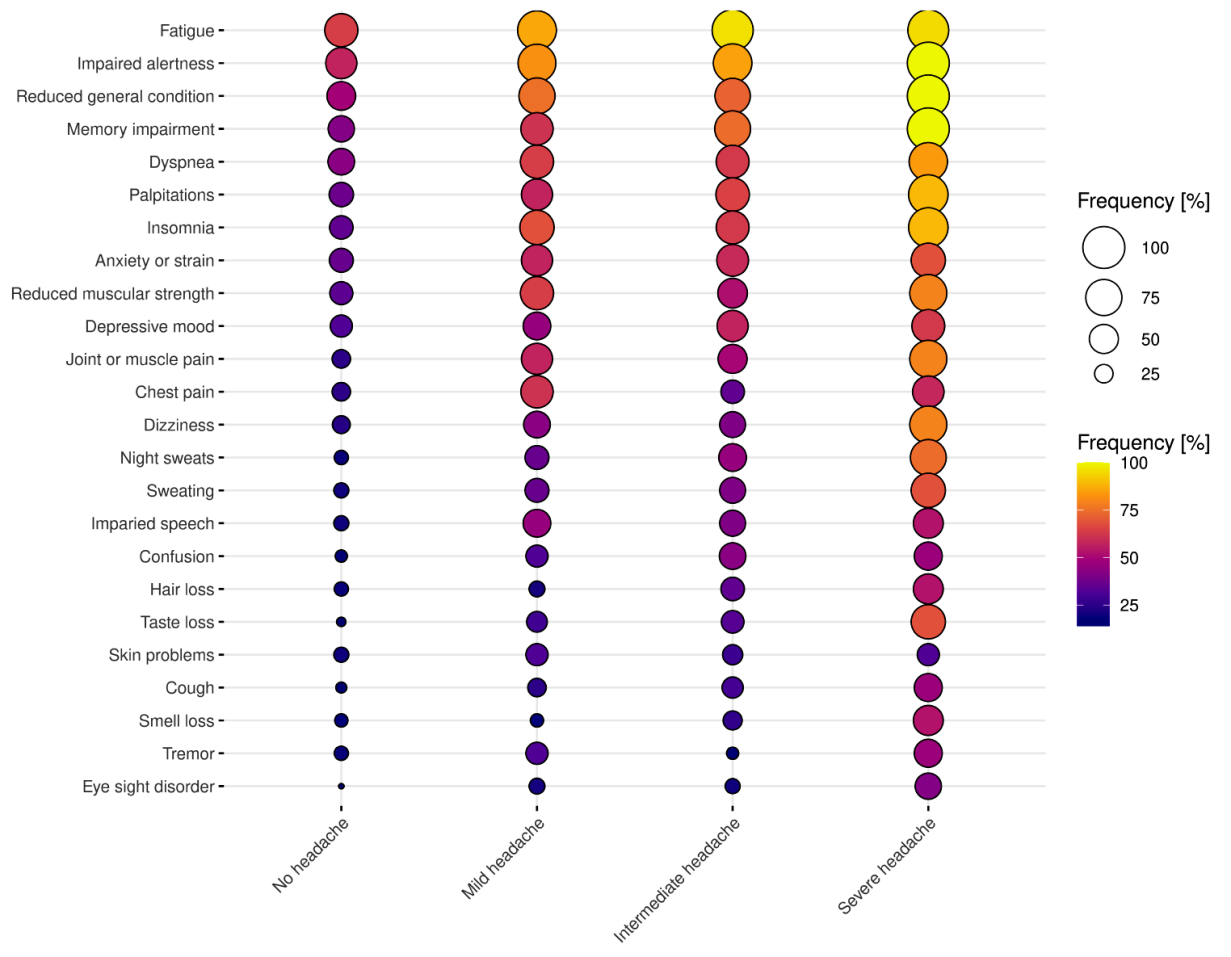
Balloon plots with frequency of symptoms across headache severity

Figure 1 gives insights into symptom frequencies across headache severity (based on four-point Likert scales) in balloon plots. Most PCC patients with mild, intermediate, or severe headache frequently reported fatigue, impaired alertness, reduced general condition, memory impairment, dyspnoea, palpitations, insomnia, anxiety/tension, or reduced muscular strength (cf. Figure 1).

**Fig. 2:**
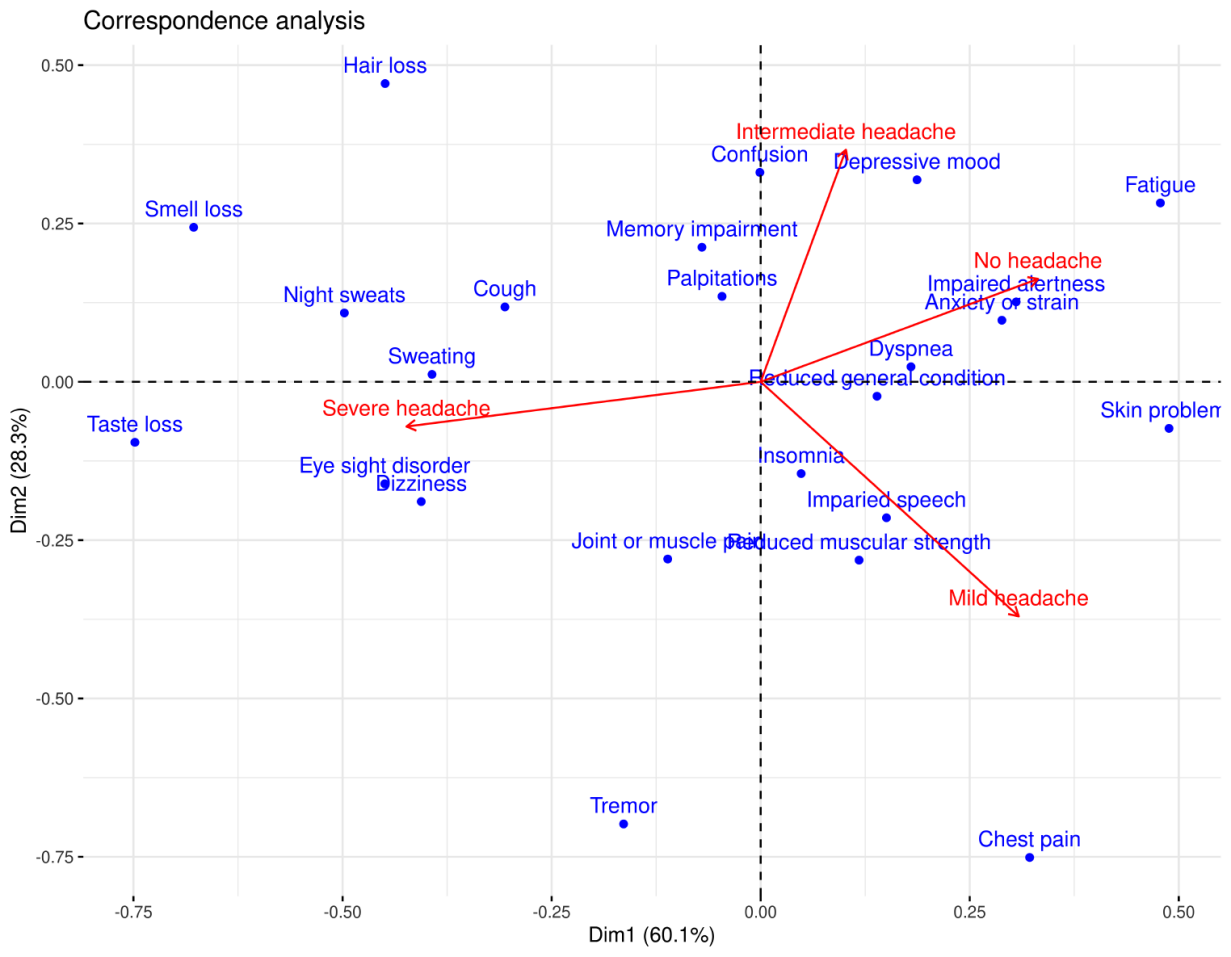
Correspondence analysis of symptoms across headache severity

Figure 2 shows a correspondence analysis of the symptoms across headache severity. The closer two symptom points are to each other in the graph, the more likely these symptoms occur together. PCC patients with no headache correlated with impaired alertness and anxiety or tension. Mild headache was associated with reduced muscular strength and impaired speech; intermediate headache with depressive mood and confusion; and severe headache with eyesight disorder, dizziness, and sweating.

## Discussion

PCC has garnered significant attention due to its diverse range of persistent symptoms that extend beyond the acute phase of the SARS-CoV-2 infection. Among these symptoms, headaches have emerged as a notable concern.

We assessed the prevalence of headache in PCC patients with PCC in a Post-COVID outpatient department at the LMU University Hospital and showed that headache occurs more frequently in PCC patients compared to the control group. However, the frequency of headaches among PCC patients is not higher than in population-based studies on headaches [39–41]. Furthermore, we identified risk factors for developing PCC associated headaches.

### Main findings

Our findings demonstrate that pre-existing psychiatric disorders are associated with headaches in PCC patients. We could not confirm that the prevalence of psychiatric disorders correlated with headache severity. However, the severity of headaches seems to correlate with the severity of depressive symptoms in the PHQ-9. This could suggest that headache, as a symptom, has such a debilitating effect on PCC patients that they develop depressive symptoms. This could be an indication that particularly PCC patients with severe headaches should be screened for depressive symptoms [42]. This could also suggest that pre-existing depressive symptoms are a risk factor for developing headaches in PCC.

Our results could not confirm the assumption of previous studies that hyposmia accompanies headache as the most common symptom [13]. We could also not show that hyposmia is more frequent with more severe headaches.

Memory impairment seems to be a key symptom of PCC patients which was also confirmed in a meta-analysis by Premraj et al. [16].

Quality of life was significantly lower in all four domains of the WHOQOL-BREF (physical health, psychological health, social relationships, and environment) compared to the control group and established reference values [38]. Our study confirms prior research that PCC with headache are associated with a poor quality of life [6, 43, 44]. Further, our results are consistent with the findings of Förster et al. that PCC patients had a significantly lower health-related quality of life, and more days of sick leave [45].

Our PCC cohort had a higher fatigue rate, according to the FSS, compared to other studies [46]. This indicates that our cohort consisted of more severe PCC patients which is understandable when considering the inclusion criteria of our study.

According to our data, it is important to recognize that post-COVID headaches often coexist with other persistent symptoms, such as impaired alertness, reduced general condition, or memory impairment. The interaction between these symptoms may indicate shared underlying mechanisms, such as dysregulation of immune responses or autonomic nervous system dysfunction [47]. Identifying these intersections could potentially reveal common targets for therapeutic interventions, offering comprehensive treatment strategies for individuals dealing with multiple Post-COVID-19 symptoms.

Diagnosing and managing PCC associated headaches present unique challenges. The lack of specific biomarkers for these headaches underscores the importance of a thorough clinical assessment, including detailed history-taking and comprehensive headache evaluation. Given the potential overlap of symptoms, clinicians must differentiate between PCC associated headaches and other primary headache disorders that existed previously and are only now intensified. Tailoring treatment approaches based on headache characteristics, patient preferences, and coexisting symptoms is paramount. Integrating pharmacological interventions, lifestyle modifications, and psychological support can provide a holistic approach to headache treatment in the context of PCC [23].

The association between PCC and headaches remains an active area of research. Further studies are needed to elucidate the precise mechanisms driving headache development in this context [23]. Longitudinal studies assessing the evolution of post-COVID headaches over time can provide insights into e.g. potential predictors of chronicity. Additionally, investigating the efficacy of various treatment modalities, such as medications targeting inflammation or neural pathways, can guide evidence-based treatment strategies. Collaboration among multidisciplinary teams, including neurologists, infectious disease specialists, and mental health professionals, is crucial for advancing our understanding of post-COVID headaches and optimizing patient care [24].

### Strengths and limitations

One strength of our study is that we correlate headaches with a broad range of interdisciplinary symptoms, thereby identifying patterns for future interdisciplinary research questions.

Various factors may influence the selection of patients referred to our Post-COVID-19 outpatient department, such as awareness of the department’s existence, health literacy, and the presence of symptoms that impact their daily activities. Our patient cohort consisted of particularly severe PCC cases that could no longer be managed by general practitioners in the outpatient setting. Participants were asked to quantify the severity of their headache. However, the quality of pain was not further classified (e.g. tension headache, migraine, trigeminal neuralgia).

The current sample size for a Post-COVID-19 study is relatively large, whereas the control group is comparatively small. Nevertheless, for some PCC symptoms, conclusions can only be drawn with limitations since the sample size remains too small. In future, a more detailed inquiry into headache frequency and the number of headache days per month would be helpful for a deeper interpretation of the data. Our sample lacks data categorized by social determinants of health, which might inadvertently omit opportunities for subgroup analyses and the exploration of potential disparities within these groups. We would like to emphasize the comparison of data and networking within Germany, the European Union, and other high-income countries. Relevant constructs, like quality of life, were assessed via self-rating scales which bear the risk of recall biases [48].

## Conclusions

Headache seems to be a relevant symptom in patients with PCC. This study highlights a descriptive analysis of headaches in the context of PCC. To improve the quality of life in patients with PCC, neurological and psychiatric symptoms in particular should be focused on. Neurological symptoms such as headaches should be assessed and treated in the context of PCC not only by neurologists but also by other disciplines and in the context of all PCC symptoms. As our understanding of PCC continues to evolve, further research is warranted to unravel the underlying mechanisms, inform diagnostic approaches, and develop effective interventions, ultimately improving the quality of life for individuals affected by post-COVID headaches.

## Electronic supplementary material

Below is the link to the electronic supplementary material.

Supplementary Material 1

## Data Availability

All clinical data will be made available upon reasonable request.

## Abbreviations

DRKS: Deutsches Register Klinischer Studien
ECDC: European Centre for Disease Prevention and Control
FSS: Fatigue Severity Scale
IL-6: Interleukin 6
IL-10: Interleukin 10
IQR: Interquartile ranges
LCARS: Lightweight Clinical Data Acquisition and Management Software for Clinical Research
LMU: LMU University Hospital in Munich
Mdn: Median values
NMDA: N-methyl-D-aspartate
PCC: Post-COVID-19 condition
PHQ-9: Patient Health Questionnaire
PRO: Patient-reported outcome
SD: Standard deviation
WHO: World Health Organization
WHOQOL-BREF: WHO Quality of Life assessment
